# Health risks in international container and bulk cargo transport due to volatile toxic compounds

**DOI:** 10.1186/s12995-015-0059-4

**Published:** 2015-05-20

**Authors:** Xaver Baur, Lygia Therese Budnik, Zhiwei Zhao, Magne Bratveit, Rune Djurhuus, Louis Verschoor, Federico Maria Rubino, Claudio Colosio, Jorgen R Jepsen

**Affiliations:** European Society for Environmental and Occupational Medicine (EOM), Berlin, Germany; Institute for Occupational Medicine, Charité University Medicine, Charité-Campus Benjamin Franklin, Berlin, Germany; Institute for Occupational and Maritime (ZfAM), University Medical Center Hamburg-Eppendorf, Division Occupational Toxicology and Immunology, University of Hamburg, Hamburg, Germany; Dalian Maritime University (DMU), Dalian, Ganjingzi China; Department of Occupational Medicine, Haukeland University Hospital, Bergen, Norway; Occupational and Environmental Medicine, University of Bergen, Bergen, Norway; Department of Global Public Health and Primary Care, Occupational and Environmental Medicine, University of Bergen, Bergen, Norway; Norwegian Center for Maritime Medicine, Department of Occupational Medicine, Haukeland University Hospital, Bergen, Norway; Expertise Centre Environmental Medicine (ECEMed), Rijnstate Teaching Hospital, AA Velp, the Netherlands; Department of Health Sciences of the University of Milano and International Centre of Rural Health, San Paolo University Hospital Milano, Milan, Italy; Centre of Maritime Health and Society, Institute of Public Health, University of Southern Denmark, Odense, Denmark

## Abstract

To ensure the preservation and quality of the goods, physical (i.e. radiation) or chemical pest control is needed. The dark side of such consents may bear health risks in international transport and production sharing. In fact, between 10% and 20% of all containers arriving European harbors were shown to contain volatile toxic substances above the exposure limit values. Possible exposure to these toxic chemicals may occur not only for the applicators but also the receiver by off gassing from products, packing materials or transport units like containers. A number of intoxications, some with lethal outcome, occur not only during the fumigation, but also during freight transport (on bulk carriers and other transport vessels), as well as in the logistic lines during loading and unloading. Risk occupations include dock-workers, seafarers, inspectors, as well as the usually uninformed workers of importing enterprises that unload the products. Bystanders as well as vulnerable consumers may also be at risk. Ongoing studies focus on the release of these toxic volatile substances from various goods. It was shown that the half-lives of the off-gassing process range between minutes and months, depending on the toxic substance, its chemical reactivity, concentration, the temperature, the contaminated matrix (goods and packing materials), and the packing density in the transport units. Regulations on declaration and handling dangerous goods are mostly not followed.

It is obvious that this hazardous situation in freight transport urgently requires preventive steps. In order to improve awareness and relevant knowledge there is a need for more comprehensive information on chemical hazards and a broader implementation of the already existing regulations and guidelines, such as those from ILO, IMO, and national authorities. It is also necessary to have regular controls by the authorities on a worldwide scale, which should be followed by sanctions in case of disregarding regulations. Further, fumigated containers must have a warning sign corresponding to international recommendations and national regulations, and freight documents have to indicate any potential hazard during stripping the goods.

**Electronic supplementary material:**

The online version of this article (doi:10.1186/s12995-015-0059-4) contains supplementary material, which is available to authorized users.

## Introduction

Transporting more than 70% of all international freight, container ships represent the majority of the c. 40,000 merchant ships that are larger than 1,000 gross register tonnes. A minor fraction of transported goods are carried by bulk carriers, tankers, reefers, roll-on/rill-off ships. All these ships may be categorized by their operations with liners running regularly and operating on a schedule and tramps without fixed runs that will go wherever a suitable cargo takes it.

Freight on board ships in containers as well as in other forms is frequently disinfected by use of toxic fumigants. All fumigants, which are used to protect the transported freight from alien species and to inhibit their spread to foreign countries comprise a potential health hazard which draws attention to, the role of exposure situation for risk assessment, especially for exposed dockers, other port staff and transport workers involved in unloading imported production parts and goods, but presumably also to vulnerable end consumers. They affect mainly the central and peripheral nervous system and the respiratory tract, but may also cause cancer. Further, recent studies identified a varying spectrum of toxic industrial chemicals released from the newly manufactured transported products (see chapter on Intoxication incidents).

Unfortunately, so far, there is limited communication on potential health risks from volatile toxic substances in globally transported freight between producing companies, logistic companies along the transport line, national controlling agencies, and the end-user. Further, common information and communication systems do not exist at all. Appropriate national and supra-national common databases comprising all relevant health information of transported freight are urgently needed. This would give full transparency, stimulate precautions and allow the reduction of time consuming, expensive and cumbersome controls and measurements. It would reduce the health risks and provide protection against intoxications.

This review aims to give an overview of the most prominent toxicants in transport containers, published intoxications, and show findings from examined cases (including case histories and exposure assessment). We suggest biomonitoring and medical examination scheme for exposed workers. The legislative framework and the practice of handling transport containers in different settings are reviewed and appropriate preventive measures are presented. Additionally a questionnaire based survey on practical handling of transport container in China (exporting country) and Denmark (importing country) were performed.

The objective of this overview was to give a state of the art presentation on endangering exposures to volatile substances in freight transport, on the associated possible adverse health effects, diagnostics, regulations and appropriate preventive measures.

### Outline

The review is divided into several paragraphs, discussing various aspects of health issues associated with international container and bulk cargo transport. The separate sections deal with the following issues.

1. Health effects of toxic substances in container transport
  - Health effects of individual fumigants
  - Health effects of preservatives and ripening inhibitors
  - Health effects of toxic substances released from cargo
2. Medical examination of subjects with suspected intoxications
  - Case report: intoxications in a storage room from off gassing fumigants
3. Practice of handling transport containers in exporting and receiving countries. Pilot studies in China and Denmark
  - Pilot study in China
  - Pilot study in Denmark
  - Discussion of the pilot studies in China and Denmark
4. Occurrences of intoxications with chemicals used for pest control in transport containers and on bulk cargo ships
  - Fumigant intoxications – general
  - Fumigated transport containers
  - Fumigated bulk cargo ships
  - Summary of incidents
  - Concluding remarks
5. Regulations on fumigants
6. Preventive measures
  - Applied preventive measures
  - Labelling
  - Knowledge and awareness
  - Guidelines for safe procedures
  - Measurements
  - Ventilation
  - Personal protective equipment

## Health effects of toxic substances in container transport

Container transport over sea has expanded enormously in the last thirty years. The use of toxic substances for fumigation and preserving (i.e. ripening inhibition) goods has increased concordantly with a tendency to decay during transport [1]. Other sources of toxic substances in container transport are the encasement material and the transported cargo itself. During the whole process of loading, transporting and unloading, people are at risk for exposure to these toxic substances. Even consumers at the end of the chain may be at risk [2]. The occurrence and severity of the health effects depend on a number of factors (see below).

### Factors determining occurrence and severity of health effects

- The concentration of the substance the subject is exposed to
- The duration of exposure
- Whether the exposure was acute and/or chronic
- The individual sensibility of the exposed subject

Additive or synergistic effects from co-exposure to the substances.

It is important to keep in mind that acute intoxications with these substances can lead to health effects that persist for months to years or even lifelong. The Expertise Centre Environmental Medicine (ECEMed) Rijnstate Teaching Hospital in Rijnstate, the Netherlands, and in the outpatient departments have primarily seen health complaints due to exposure during unloading containers or drivers, who had to open the container in order to drive their truck to the dock for unloading [2,3]. In this chapter we will briefly discuss the health effects of the most common toxic substances people are exposed to in container transport (see Table 1).

**Table 1:** **Major toxic substances in container atmosphere**

**Fumigation**	**Preservation**	**Encasement**	**Cargo**
**Phosphine**	Carbon monoxide	Formaldehyde	Benzene
**Bromomethane (methyl bromide)**	Carbon dioxide		Xylene
**Sulfuryl difluoride**			1,2-Dichloroethane
**Formaldehyde**			Ethylbenzene
**Ethylene oxide**			Toluene
**1,2-Dichloroethane (ethylene dichloride)**			Dichloromethane
**Dichloromethane**			Other organic solvents
**Chloropicrin (trichloro(nitro)methane)**			

### Health effects of individual fumigants

#### Phosphine

Phosphine is used especially in the transport of grain in containers as well as as bulk carrier transport in trains or ships. Tablets containing aluminium- or magnesium phosphide are combined in small sockets or long sleeves. The phosphide reacts with the moisture in the air of the container to produce phosphine. Mostly, the containers are holding silica gel for drying the container in order to prevent too much formation of phosphine during the transport. Thus a crucial moment is the opening of the container. The remaining phosphide starts forming phosphine when humidity enters the container from outside. The same reaction takes place for loads in the holds of ships at the presence of remaining phosphide. Especially this moment leads to acute intoxications. After this acute moment continuous exposure may take place due to the absorption of phosphine to the clothes of the person.

In the western world, intoxications were previously seen primarily in employees of phosphine producing plants and with agricultural use. In Asia phosphine is reported as a suicidal agent with death rates as high as sixty percent [4].

Phosphide reacts with moisture (i.e. water) to form gaseous phosphine [5]. Phosphine interferes with the electron transport chain in the mitochondria [6,7]. Already within one minute after exposure, effects on the respiratory and gastrointestinal tract are found. Respiratory symptoms are irritation of the (upper) airways leading to pain and cough, shortness of breath and eventually hypoxia and a feeling of severe pressure on the thorax. The gastrointestinal symptoms are nausea, abdominal discomfort, diarrhea and vomiting. Neurological and skeletal muscle effects occur rapidly thereafter. Involvement of the central as well as the peripheral nervous system lead to dizziness, disorientation, headache, neuropathic pain, mainly in arms and legs, and eventually decreased consciousness and coma. All skeletal muscles can be involved with severe pain and loss of tonus. Single acute exposure leads to muscular damage with a peak after 24 to 48 hours after the acute incident and lasting for weeks to months. In severe intoxication, the cardiovascular system is also involved. Cardiac arrhythmia, hypotension, hypoxia and renal failure ultimately lead to death [8]. The majority of patients that come to the attention of doctors are seen at the emergency room in general hospitals. Due to unfamiliarity with the clinical syndrome, the correct diagnosis is often not made and patients are discharged from the emergency room under the diagnosis of hyperventilation syndrome. Measurement of PaO2 and oxygen saturation can rapidly distinguish between hyperventilation and phosphine intoxication, the latter showing decreased values [9].

#### Methyl bromide (bromomethane)

Since 1938 methyl bromide has been used widespread as an agricultural fumigant. During the last twenty years there has been a rapid rise in the use of methyl bromide for fumigation in transport containers [10].

The Montreal Protocol on Substances that Deplete the Ozone Layer (http://ozone.unep.org/new_site/en/montreal_protocol.php) has put an end to the broad use of methyl bromide in agriculture in order to prevent further reduction of the ozone layer caused by halogenated methyl/ethyl bromides and –chlorides (i.e. methyl bromide/bromomethane). Due to its toxicity, the use of methyl bromide in the European Union has been forbidden since 2010, but exceptions are made, especially with regard to the International Standards for Phytosanitary Measures 15, ISPM 15 (https://www.ippc.int/publications/regulation-wood-packaging-material-international-trade-0). Consequently, the use of methyl bromide for fumigation of stow wood in sea containers remains allowed until 2015 (and continues to be used, if no alternative product is available, as a part of CUEs, critical use examples). The increasing use as a fumigant in sea container (a part of the CUE) transport leads to exposure to methyl bromide of a new group of workers, handling import containers.

28 fatal cases of methyl bromide intoxication [10] have been described in the literature [11]. In 1983, this has risen above 950 fatal cases [11]. Acute intoxication is characterized by early symptoms like headache, vomiting, sore throat, vertigo and visual disturbances, followed by reduced consciousness, coma and convulsions [10,12]. Chronic effects can last for years and are confined mainly to the central (chronic toxic encephalopathy) and peripheral nervous system (painful neuropathy) together with general complaints of fatigue [13]. See Table 2 for limit values.

**Table 2:** **Health based occupational exposure limits values for major fumigants**

**Fumigant**	**Acute effects/workplace**	**Carcinogenic effects (potential)**
**Chemical**	**Occupational exposure limit, OEL [mg/m** ^**3**^ **]**	**Carcinogen = + (Class)**
**Phosphine**	**0.14**	**-**
**Bromomethane (methyl bromide)**	**3.89**	**+ (3)**
**Sulfuryl difluoride**	**20.9**	**-**
**Formaldehyde**	**0.37**	**+ (4)**
**Ethylene oxide**	**1.80**	**+ (2)**
**1,2-Dichloroethane (ethylene dichloride)**	**4.05**	**+ (2)**
**Dichloromethane (chloromethane)**	**86.75**	**+ (3)**
**Chloropicrin (trichloro(nitro)methane)**	**0.672**	**-**

Sources: NIOSH; National Institute for Occupational Safety and Health, Center for Disease Control and Prevention (www.cdc.gov/niosh/npg).

IRAC, International Agency for Research on Cancer (http://monographs.iarc.fr/ENG/Monographs/vol100F/mono100F.pdf).

(see [19] for further details on community exposure levels and thresholds).

#### 1,2-Dichloroethane (ethylene dichloride)

Acute exposure to 1,2-dichloroethane (ethylene dichloride) leads to headache, dizziness and hypoxia. Chronic exposure elicits chronic toxic encephalopathy (see organic solvents) and is frequently accompanied by liver function tests [14]. See Table 2 for limit values.

### Sulfuryl difluoride (sulfuryl fluoride)

Sulfuryl difluoride is considered as a less toxic substitute for methyl bromide. However, it does have ozone-depleting as well as toxic effects including acute and chronic encephalopathy, disturbances in the olfactory function, and neuropathy with reduced nerve conduction velocity [12]. See Table 2 for limit values.

### Formaldehyde

Acute intoxication is characterized by dizziness, nausea and vomiting, fatigue, feeling of drunkenness, disturbance in coordination and concentration followed by headache, malaise, hypersensitivity for noise and light, and upper airway symptoms like cough, shortness of breath, a hoarse voice and sometimes skin eruptions. Symptoms and complaints last for days or weeks [15].

More than 90% of inhaled formaldehyde is taken up in the upper airway system and leads to local irritation and neuro-cognitive effects. Severe exposure may cause additional complaints from the lower respiratory tract like shortness of breath, increased sputum production and coughing.

Formaldehyde may also impose severe long-term effects since it is carcinogenic to humans [16].

### Chloropicrin (trichloro(nitro)methane, nitrochloroform)

This previously used fumigant may be added as an indicator to (odourless) methyl bromide or other fumigants due its strong odour. See Table 2 for limit values.

### Health effects of preservatives and ripening inhibitors

#### Carbon monoxide

Exposure to carbon monoxide leads to non-specific complaints like headache, dizziness, somnolence to unconsciousness, flu-like symptoms, and seizures. If such symptoms occur in the context of handling containers, especially with fruits, one should be alarmed. The primary measures to be taken are immediate removal of the victim from the site and dispense oxygen. Omitting these measures can eventually lead to death. Since carbon monoxide has a much higher affinity for hemoglobin than oxygen, severe hypoxia rapidly develops [17].

#### Carbon dioxide

Carbon dioxide, frequently combined with reduced oxygen levels, is added to sea containers with fruits as cargo in order to prevent ripening during transport. It acts as an asphyxiant and in addition has toxic effects at the cellular level.

Low concentrations (up to 5%) lead to headache, dizziness, sweating and shortness of breath. Concentrations up to ten percent cause hyperventilation, tachycardia and worsening of headache and dizziness with laboratory findings of metabolic acidosis. Severe intoxications occur at concentrations above 10% with additional symptoms of drowsiness, muscle twitching and loss of consciousness with severe hypoxia. Higher concentrations elicit convulsions, coma and death. Immediate removal of the person from the area of exposure should be done along with administration of oxygen and general life support [18].

### Health effects of toxic substances released from cargo

#### Organic solvents (benzene, xylene, ethyl benzene and toluene, chloromethane)

All kinds of organic solvents and degreasing agents fall into this category. These solvents are emitted from goods such as furniture or shoes that have been manufactured with the use of paint, lacquer or glue. Given the long duration of transport over sea in a closed container (usually without ventilation), high concentrations may build up [19]. As soon as the doors are opened people entering the container may be exposed to high concentrations of solvents.

All organic solvents give rise to acute and chronic toxic encephalopathy (CTE) the severity of which depends on the amount and duration of the exposure and the presence and amount of peak exposures [20]. Acute neurotoxic effects resemble alcohol intoxication with comparable narcotic features like dizziness, disorientation, euphoria and a feeling of drunkenness [21]. Chronic effects are seen after exposure duration of up to 3 – 10 years and are categorized in stages I to III (WHO, see Table 3).

**Table 3:** **Classification of CTE according to the WHO**

	**Symptoms**	**Cognitive defects**	**Neurological abnormalities**
Class I	Fatigue, diminished memory, concentration and initiative	No objective dysfunction	No objective abnormalities
Organic affective syndrome	Change in personality. Poor impulse control, lowered mood and motivation, irritability, anxiety, emotional lability		
Class II	Difficulties in concentration and attention, impairment of memory, decrease in learning capacity	Objective evidence of cognitive impairment	Minor neurological signs
Mild chronic toxic encephalopathy			
Class III	Marked global deterioration in intellect and memory	Marked global deterioration in intellect and memory	Neurological signs and/or neuroradiological abnormal findings
Severe chronic toxic encephalopathy			

In the case pressed wood panels are used, formaldehyde is often emitted from the panels during transport. See for health effects of formaldehyde in the section on fumigants.

In addition to the neurotoxic effects benzene is also carcinogenic to humans (IARC Monographs, 100 F, 2012). Accumulating evidence indicates effects on several blood cell types at ever lower concentrations, suggesting risk of developing malignancies of the hematopoietic system [22-24].

## Medical examination of subjects with suspected intoxications

As mentioned, acute or chronic intoxications by fumigants or toxic industrial chemicals are typically associated with non-specific symptoms and therefore frequently misdiagnosed. The consequence may be ongoing hazardous exposures in the workplace with serious and frequently non-reversible disorders. This requires that workers as well as physicians are aware of these potential dangerous exposures at the workplace.

The crucial initial step is the detailed occupational and clinical history, which should be taken by an experienced physician who is aware of potential risk exposures to volatile toxic substances in the workplace (see dignostic set-up below). A well-known detailed questionnaire is available from Safe Work Australia [25]. Based on the literature as well as on our own clinical experience we have developed a new comprehensive questionnaire (FumEx), which is now available in three languages [26]. It was shown in various applications to be very useful in field studies as well as for individual intoxication cases. It is not too time-consuming and it is easy to handle by the patient by adding information by the physician performing the crucial personal interview. FumEx allows to settle/fix the time, the duration and the type of workplace exposures [27], the applied protective devices, and to detect acute and chronic symptoms related to workplace exposures and to exposures outside the workplace. For the full diagnosis, the work history is followed by physical examination and functional tests that reflect the potentially affected organs such as lung function testing, blood analyses, and neurological and neuropsychological investigations [28,29]. Abnormal findings may require sophisticated examinations by other medical disciplines such as neurology or pneumology. Imaging such as MRT or cranial CT may also be indicated. Early starting of biomonitoring may help to identify the causative toxic substance. Unfortunately, there is no specific therapy and no available antidote; only symptomatic treatment is possible.

**Table 4:** **Examples of reported incidents/intoxications with fumigants in transport containers (see Table in attachment for more detailed case reports)**

**Date**	**Location**	**Fumigant**	**No. of subjects**		**Reference/source**
			**Affected**	**Fatalities**	
Nov. 2008 2008 - 2006	-	PH_3_	1	0	Case reports [28] (PubMed)
	-	PH_3_?	1	0	
	Warehouse, Germany	1,2-dichloroethane	2	0	
2006	Rotterdam	CH_3_Br	2	0	[39] (PubMed)
2008-2009	Rotterdam	1,2-dichloroethane	20	0	[40] (Google)
		PH_3_	9	0	
		CO_2_	2	0	
		CH_3_Br	2	0	
2007-2008	The Netherlands	CH_3_Br	2	0	[42] (Web of Science)

### Immediate diagnostic set-up, which requires an experienced physician in case of assumed intoxication is outlined below (see also the questionnaire in Additional file 1)

- **Diagnostic set-up in case of suspected intoxication**
- ***Case history, questionnaire***
  - headache, dizziness, forgetfulness, concentration disorders, feeling impatient or depressed, sleepiness, emotional problems, shortness of breath, diarrhea, change of sense or taste, loss of sexual interest etc.
  - Fum-Ex2 Questionnaire is accessible online at: www.EOMsociety.org
- ***Physical and neurological examination***
- ***Lung function testing***
  - spirometry, gas exchange measurement, methacholine challenge test, spiroergometry
- ***Clinical chemistry/Blood tests:***
  - liver enzymes, muscle enzymes
  - intoxication markers
  - bromide and fluoride in serum and urine
- ***Human Biomonitoring***
  - residual fumigants in blood and urine (methyl bromide, ethylene oxide, ethylene dichloride, chloropicrin, methylene chloride, co-exposure to solvents like benzene, toluene, xylene etc.; headspace tubes – Gas-Chromatography-Mass-spectrometry analyses
  - hemoglobin adducts in blood (methylvaline and hydroxyethyl valine)
- ***Sniff sticks (olfactory test)***
- ***Colour vision test***
- ***Comprehensive neurological and neuropsychological tests, e.g.***
  - physical performance (grooved pegboard and finger tapping test)
  - test of crystallized and fluid intelligence
  - information processing speed
  - sel***e***ctive attention/concentration tests, divided attention
  - sustained attention/vigilance
  - learning/memory tests
  - decision making and pre-morbid verbal intelligence
  - logical thinking and abstraction
- ***MRT, cranial CT***

### Case report: intoxications in a storage room from off gassing fumigants

In a medium sized European company, which received electronic production parts from south east Asia and South America, several employees developed signs of intoxication on three occasions within two years [30]. Six storage room workers were exposed to fumigants off gassing from shipped products. They were unloading approximately two overseas shipments a week at the time of the incident. They all worked 8 hours per day day in a storage room. Patients 1,3,4,5 and 6 unloaded the delivered items on a regular basis, and unpacked wooden pellets with paper boxes covered with plastic. Patient 2 supervised the working place. The workers were responsible for unloading and distributing the delivered items into several separate areas on different floors. Following each incident, the workers (patients 1, 3, 4, 5 and 6) noticed itchy skin, very red eyes and suffered from recurrent epistaxis, headaches and intention tremor. After the second incident, four individuals (Pat. 1, 3, 4 and 5) additionally complained about paresthesia (pins and needles in the legs), dizziness, breathing difficulties and increasing irritability. After the third incident, patient 4 was on sick leave for several weeks; patients 1 and 3 developed immediate epistaxis with a severe headache [30]. Air samples taken in the space between the packed electronic parts with trapped gas residues (in the breathing zone of the workers) showed presence of ethylene oxide and methyl bromide. Patients (n = 6, aged from 32 to 54 yrs.) were compared with a healthy control group (n = 30, with median age of 40 yrs.). Patients underwent standardized medical examination for the symptoms of fumigant intoxication as described earlier [31,27,30]. No significantly elevated bromide levels in any of the analyzed serum samples were detected. However, we detected low methyl bromide levels (0.24 μg/L) in the blood of patient 5 collected five days after the second incident (BM2). No increased levels of ethylene dichloride, chloropicrin, methyl iodine or dichloromethane were detected in the blood samples (all were below the limit of detection). Patients 1, 3, 4, 5 and 6 showed slightly increased levels of ethylene oxide in blood and two patients displayed elevated methanol levels, Patient 5 showed presence of small amounts of methyl bromide in blood [30]. In-vivo dose monitoring by means of adducts to macromolecules after exposure to methylating agents showed elevated levels of hemoglobin adducts: methylvaline and hydroxyethylvaline. The monitoring of hemoglobin adducts additionally allows to monitor past exposures and to generate extrapolated data that might enhance the risk assessment strategy. We have calculated the theoretical exposures to methyl bromide and ethylene oxide at the time of the individual incidents. Based on these data, the intoxication could be confirmed for three patients out of six (patients, 3, 4 and 5); data for patients 1, 2 and 6 did not allow any extrapolation. The clinical examination data from these patients (data not shown here) support the evidence of intoxication. No intoxication could be confirmed for patients 1, 2 and 6. Patient 2 supervised the other workers and after the first incident patients 1 and 6 were mostly engaged in other areas of the storage room with less direct contact with (newly delivered) unloaded products. Notably, after the first accident the company reorganized the storage area with a separate zone for unpacking the incoming shipment. As reported, the patients showed serious intoxication symptoms with itchy skin, very red eyes, dizziness, breathing difficulties and increasing irritability. They suffered from headache, acrotaxia, (intention tremor) paresthesia, and/or developed immediate or recurrent epistaxis. Our case studies indicate a need for an early and comprehensive prevention system including dedicated rules for the occupational hygiene and biomonitoring procedures [30].

## Practice of handling transport containers in exporting and receiving countries. Pilot studies in China and Denmark

This section addresses the local interpretation and implementation of international and national regulations for safe container handling in China (as exporting country) and Denmark (as importing country). This is found of relevance since the involved workers may not always sufficiently know the legal regulations and the required precautions that may consequently not be properly integrated in the daily occupational health practises.

### Pilot study in China

With more than 30 years economic reform, the Chinese government is now paying more attention to the working conditions and health and safety of workers than before. However, little notice has been paid to these issues in the field of container fumigation in China [31].

Firstly, the operation of the fumigation of export containers in China is discussed in general, mainly considering the following two questions: who is operating the business and how work has been organized. It also looks at the working conditions and the health issues of workers. The research draws on a case study of a Chinese state-owned company, established in 2007, operating the fumigation of containers. It is a subsidiary company of the Provincial Administration of Inspection and Quarantine. Qualitative methods are utilised, with two first line managers being interviewed extensively and four workers being group-interviewed under the ‘supervision’ of their managers. The presence of the managers may prevent workers from expressing their concerns to some extent and hence weakens the analysis. However, available data still provide a meaningful explanation as to what is going on within that particular field.

In China, the business of fumigating export containers is under the administration of the General Administration of Inspection and Quarantine of the P.R. China. This government agency also formulates relevant policies and regulations, such as ‘Measures for Supervision and Management of Fumigation and Disinfection’ and ‘Rules of Operating Container Fumigation’. In each province, there is Provincial Administration of Inspection and Quarantine responsible for implementing the regulations and policies. The provincial agency sets up local subsidiary companies that monopolize the business of fumigation. The interviewed managers introduced:

*“Only the subsidies can operate the fumigation of exported containers. Other companies are not allowed” (interviewed manager No.2)*

The monopolization of the business of fumigation by the subsidiary companies is a result of special supports of the provincial agency to the subsidies. Firstly, the subsidies obtain credentials to provide training to the workers and issue them professional certificates. More importantly, the subsidies obtain credentials of operating fumigation and issues official certificates to the fumigated containers, which are highly recognized by foreign consignees. None of these credentials can be obtained by any other company. Last but not least important, these subsidies are supported financially by their parent agency. By monopolizing the business, the subsidies are faced with little competition in the market and do not have to concern much about lowering down the costs.

This may have some positive implications to the working condition of workers who seem very happy with their jobs. The workers in the case study received professional training and are issued nationally recognized certificates at the end of the course. They are very familiar with the operation of fumigation and operate fast. Two workers can finish the fumigation of one 20 feet container in less than 5 minutes. They work at the ‘Specified Fumigation Area’ and wear working suits, masks, and gloves during fumigation. They know the existence of health and safety regulations of fumigating and said they learnt them at the training course but could not remember them specifically. They know that they use methyl bromide for fumigation and that it is highly toxic. They are not sure whether the masks are dust masks or gas filter masks. However, they seem pretty sure that their masks can prevent them from any harm. They seem fearless. They do not report any symptoms that might be related to chemical exposure or any diseases that might be related to chemical exposure. They said they are very satisfied with their work:

*“Although there are many detailed instructions about the operation, for instance the environmental factors such as wind direction that can influence the operation, in practice it is rather straightforward – nothing complicated at all”*

*“No exposure to the chemicals”. “No danger to health”*

*“I am very happy with my job”. “I think it is a good one”. “Yes, I am happy with it”*

However, this may not be the whole story because according to the managers, the fumigation job is not a good at all. The managers said:

*“I do not think it is a good job. At least, it is not decent and it does not pay much.” (Manager No.1). “Honestly, it is the peasants (land man) who are doing the job. Even the peasant workers are not willing to do the job. It is a simple, but NOT a very good job!” (Manager No.2)*

Most of the workers in the company are peasant workers. They are temporarily employed, offered low wages, have little job security, are not provided with any social insurance and most of them received little education from school. The managers explained:

*“When there is a need, the workers are called to work” (Manager No.1). “Peasant workers are not under the social insurance scheme. Before the economic reform, workers were provided with food subsidy and work protection subsidy. But now, there is nothing. If the peasant workers want to do the job, they do it. If not, they can just leave.” (Manager No.1)*

These peasant workers are specifically trained for fumigating containers, without many other skills. They are in a very weak position and depend on their managers for job opportunities. Because of the presence of their managers, it is possible that workers did not thoroughly express their concerns about their working condition or occupational health issues, especially the problems. However, the limited alternatives of their jobs in the labour market can be another important explanation as to why the workers express their content with their current jobs.

In conclusion, the study suggests that peasant workers are in lack of job security and basic protection, especially concerning the social insurance aspect. Their weak position due to the lack of protection and support from the society is an important issue that needs addressing. It is also important to notice that fumigation of export containers is monopolized by subsidiary companies of the Provincial Administration of Inspection and Quarantine. Without much pressure for high profits, state-owned subsidies provide relatively satisfactory working condition to their workers.

It was also seen from the case study that since the European countries improved the inspecting standards of the levels of methyl bromide in the imported containers, fumigation using toxic chemicals has been gradually replaced by heat treatment in this state-owned company. This might imply that a stricter regulation may reduce the use of using toxic chemicals in this industry and improve workers’ health and safety standard.

### Pilot study in Denmark

The practice of container work was investigated in a series of semi-structured interviews with key informants including managers and safety representatives of two organizations the employees of which may be chemically exposed while handling containers [31].

Two major working populations are potentially exposed to hazardous chemicals when handling containers in Danish harbours: workers who unload freight and government officers in charge of inspecting transported goods. The practices and perceptions of workers was assessed by approaching the staff of the custom office and a container logistics company both located in Aarhus – the largest Danish container port. The selection of respondents was based on a purposeful snowball sampling method of individuals that were rich in information and experiences related to the topic. The interviews followed a template that included questions about work activities, potential chemical exposures, experienced health effects, health and safety regulations and applied preventive measures. A semi-structured face-to-face method was used to support the respondents to share their views and to gain in-depth information about the topic.

The approaches to prevention were quite different for the two populations. Group policies in the logistic company aimed to follow the vision for conducting business in a safe manner and to comply with applicable laws and regulations. The safety instructions were structured and easily available but had no stated relation to national and international regulations. In case of suspicion caused by the presence of a fumigation certificate, labeling as “dangerous goods” or closed container ventilation valves, ventilation, filter mask and gloves were prescribed. The custom office had safety instructions applying to control of containers, storage and warehousing that relate to the IMDG code, to European and Danish directives as well as to a chemical database established by the Danish occupational health service for seafarers (Seahealth).

In spite of limited knowledge about the potential health risks from exposure to fumigated containers, all the interviewees agreed on their existence. They expressed concern about frequent chemical exposure and estimated the proportion of polluted containers to be high, up to 50%. According to their experiences, 20% to 50% of the containers have a distinct perceivable odour. In contrast, they recalled that few containers were documented and labelled as previously fumigated or chemically treated. Methyl bromide was the only fumigant encountered in practice that all interviewed persons could quote. Some interviewees expressed their awareness about general industrial chemicals and noted experience with signs of exposure, such as a white powder in some containers, but could not specify the chemicals.

Most interviewees assumed that chemical exposures from transport containers may chronically damage workers’ health. Inspectors were increasingly aware of the risk, but had very limited specific knowledge. Manual workers were also rather unknowledgeable about the problem and fear was not typical among them.

Headache as the most commonly reported acute health effect was more frequently experienced in the past and tends to be related to special types of freights. Eye irritation and unspecified discomfort are also experienced occasionally. However, no interviewees recalled chronic intoxications with medically established connection to previous chemical exposures.

The interviewed managers were aware of the existence of relevant international and national regulations although they were typically not able to specify them. On the other hand, the interviewees were more knowledgeable about the local instructions of their organisations. Besides the general provisions for workplace health and safety, e.g. risk assessment, education, use of safety equipment and personal protective equipment, the local instructions, however, give insufficient guidance for several practical challenges of preventing chemical exposures during work with containers.

In practice, the preventive measures are primarily based on the attempt to identify the containers that carry occupational health risks. Although the review of documentation was reported to be the main way to get information about previous chemical treatment it was rarely seen and likely to be absent in a major proportion of fumigated containers. Apart from the documents, strange odour, the type of goods and the country of origin may create suspicion. Containers arriving from developing countries with food, footwear, furniture and other wood products were most frequently regarded as carrying chemical hazards. Equipment for monitoring of container air is available in some of the organisations only, but even if available, it can detect only methyl bromide but no other potentially harmful chemicals. In some organisations a suspicion is sufficient for applying specific preventive measures while others would require positive results of air measurements to take action.

The decision and choice of preventive measures to be used in practice tend to be left to the directly involved employees and the actions taken show considerable variation. The main preventive measure to reduce chemical exposure is passive ventilation; active ventilation is not applied in practice. The conditions of aeration are not consistently prescribed. The applied ventilation times vary between 2 and 48 hours without knowledge of whether this amount of time is sufficient. The other major way of preventing adverse health effects of chemical exposures is the use of personal protective equipment. Dust masks are usually provided to the workers, but do not prevent passage to the airways of chemical vapours. Gloves are typically provided and the use of coveralls reported by some interviewees. In practice, however, the overall frequency of wearing personal protective equipment is low.

### Discussion of the pilot studies in China and Denmark

Both presented experiences from China and Denmark are likely to represent “best cases”. In China, the state monopoly of fumigation without competition permits a certain level of training and protection of workers. Even heat treatment was used when adequate. Still, the peasant workers are disfavoured by the absence of social security. The importing countries experience adequate labelling as being rare even when most containers arrive from China. In Denmark, the set standards aimed to provide full protection. Still, monitoring and ventilation practices and e.g. the use of respiratory protection were clearly inadequate. We have little information about fumigation practices elsewhere in exporting countries, but the level of protection might well be inferior compared to China. Denmark has relatively high standards relating to chemical exposure at work, and exposure levels may well be much higher in other importing countries with lower standards of protection. Some Incidents of intoxications with chemicals used for pest control in transport containers and on bulk cargo ships.

## Occurrences of intoxications with chemicals used for pest control in transport containers and on bulk cargo ships

### Fumigant intoxications – general

Incidents with fumigants used for pest control are reported frequently [32-35]. A lot of cases occur in the agricultural use of pesticides, and in some cases affecting both applicators and people living in the neighbourhood [36]. Some incidents occur in relation to fumigation of buildings, both public buildings and private homes [37,38]. A recent incident was reported in in January 2014 where a private home in Jerusalem, Israel, was fumigated with phosphine by a professional exterminator, and two children died. Two other children were seriously harmed but survived.(http://www.jpost.com/National-News/Family-of-6-believed-poisoned-by-pesticides-in-Jerusalem-home-toddler-dies-338999).

### Fumigated transport containers

Although adverse health effects after occupational exposures to pesticides seem to occur frequently, the documentation of these incidents are often insufficient, lacking traceable description of exposure with respect to site and time, well documented exposure data, adverse health effects, clinical symptoms and not at least: publication of the incidents/cases in well-respected and traceable sources.

In particular, incidents related to opening of transport containers or fumigant intoxications on bulk cargo ships are seldom reported in scientific journals or described in public available reports. For those that are reported, the sources are variable, ranging from a few scientific papers, conference presentations to newspaper articles and short notes in organisations like Marine Accident Investigation Branch (MAIB).

To address container incidents a literature survey was conducted on PubMed in October 2014 with the following search string in the Title/Abstract field: "(toxic* OR poison* OR intoxic*) AND (fumiga* NOT fumigatus) AND container*". The search returned 43 references. Nearly 30% of all papers regarding fumigants or hazardous volatile chemicals in transport containers were by Baur and various co-partner, who started to collect the cases (see Table in Additional file 2). The PubMed search revealed only one paper with sufficient documentation of an intoxication case related to opening of transport containers: Two dock workers who opened a container in the port of Rotterdam in 2006 were acutely intoxicated, and subsequent field analysis by the fire brigade confirmed the presence of methyl bromide as the likely causative agent, although the paper did not contain any data showing the methyl bromide levels [39].

Two papers reported exposure incidents in detail. Preisser et al. described 26 patients referred to a clinic in Germany with symptoms of pesticide intoxication after opening of transport containers [29]. The authors were able to confirm the diagnosis based on typical symptoms and extensive clinical examination. Additionally by laboratory analysis ethylene dichloride, methyl bromide, phosphine and methylene chloride were identified. The predominant symptoms were headaches, concentration and memory problems, dizziness and nausea, irritation of the skin and mucous membranes and a reduced ability to do exercise. In addition to the neurological and neuropsychological impairments the analyses verified the development of reactive airways dysfunction syndrome (RADS) in 14 of 26 patients with long lasting symptoms due to their contact with fumigants.

Later Budnik et al. has performed more comprehensive exposure assessment using biomonitoring data for 164 subjects exposed to halogenated hydrocarbon pesticides (methyl bromide and ethylene dichloride) [27]. Another paper described 33 cases of presumed intoxication by transport containers [40]. The cases indicated phosphine and ethylene dichloride as the fumigants. However, the paper lacked detailed documentation of exposure data and for two of the cases there were no information of where the incident took place. A recent case report documented phosphine intoxication on board of bulk carrier in France [41].

Similar search on Web of Science added a single reference; this was a meeting abstract presenting a case report with two dock-workers being poisoned by methyl bromide as they opened a transport container. The abstract did not contain any information of location and date, but the incident most probably occurred in the Netherlands since the authors were from the National Institute for Health and the Environment in the Netherlands. No exposure data were included [42]. A Google search reveals two short notes describing patients referred to the Expertise Centre Environmental Medicine in Arnhem, The Netherlands after intoxication by container fumigants [9,40].

### Fumigated bulk cargo ships

Incidents related to transport of bulk cargo on ships seem to be slightly more frequently reported in relevant publications, e.g. as Safety Flyers/Accidents Flyers by MAIB internet site (www.maib.gov.uk). A similar, systematic literature survey as for container contamination was conducted for fumigation on bulk cargo ships. A search on PubMed in October 2014 with the following search string in the Title/Abstract field: "(toxic* OR poison* OR intoxic*) AND ship" returned 223 references. Replacing "ship" with "freighter" gave 3 additional results. Another search with the string "(fumiga* NOT fumigatus) AND (ship OR crew)" in the Title/Abstract field returned 21 references. The search results identified 3 scientific papers describing incidents on board bulk cargo ships: One on a Polish ship in Vietnam, 1967 [43], one on a grain freighter on the Canadian/American East Coast in 1978 [44], and a survey on Danish merchant ships 1988–1996 carried out by the Institute of Maritime Medicine and Danish Maritime Authority in Denmark [45,46]. The latter investigation [45] showed 6 intoxication incidents due to fumigation of cargo with pesticides, 4 of these were fatalities. The oldest publication was not assessed due to difficulties in retrieving the paper.

The PubMed search was then supplemented with searches for incident reports on the web sites of International Labour Organization (ILO), the International Maritime Organization (IMO, the International Maritime Health Association (IMHA), Marine Accident Investigation Branch (MAIB), and the international maritime insurance companies Gard, Skuld and Norwegian Hull Club. The two latter companies did not have any incident reports made public, but Gard described several incidents in Gard News 204 (November 2011/January 2012). Neither ILO (http://www.ilo.org), IMO (http://www.imo.org) nor International Maritime Health Association had any publications on incidents/case reports. MAIB showed two incidents in the period from 1999 to date: One fatality with phosphine on a bulk cargo ship loaded with grain in 2007 was released by MAIB as an Accident Flyer. Another hazardous incident happened by unloading a maize cargo in 2012 without any serious injuries was released by MAIB as a Marine incident report 21/2013 (MAIB, 2013; http://www.maib.gov.uk/publications/index.cfm).

The majority of reported cases are bulk transport of foodstuffs like grain, flour etc., and nearly all of these are treated with aluminium or magnesium phosphide in solid form. The phosphide then reacts with water (as moisture in the air or in the cargo) and releases phosphine (PH_3_) gas as the active pesticide. A unique feature of this way of administrating the fumigant is that the moisture/water may be a limiting factor and cause the reaction to stop, leaving solid phosphide in the cargo. When it arrives at the destination and the holds are opened, replenishment with ambient, moist air may restart the reaction liberating phosphine gas.

### Summary of incidents

A brief summary of the incidents with fumigated containers and bulk cargo ships that have implied health effects on humans are shown in Tables 4 and 5, respectively (see also Table in Additional file 2 for more detailed list of incidences). The majority of cases consist of dock workers and seafarers. However, the incidents with bulk cargo ships also include some stowaways. The Tables show the reported incidents with approximate location, date, number of affected subjects, the number of fatalities and the sources of information.

**Table 5:** **Examples of reported incidents/intoxications with fumigants on bulk cargo ships**

**Date**	**Location**	**Fumigant**	**No. of subjects**		**Reference/source**
			**Affected**	**Fatalities**	
September 1978	Bulk grain freighter, East coast Canada/USA	PH_3_	31^1)^	2^1)^	[46] (PubMed)
1988-1996	Danish bulk carriers	PH_3_	2	1^2)^	[45] (PubMed)
		Unknown pesticide	4	3^2)^	
October 2007	General cargo ship, Russia – UK	PH_3_	1	1	MAIB Accident Flyer 1/2008
2010	General cargo ship, Latvia – Antwerp	PH_3_	2	1	Gard News 204 Nov 2011/Jan 2012 http://www.gard.no/
2010	Bulk carrier US East coast	PH_3_	16	0	
2009	General cargo ship, Lagos, Nigeria	PH_3_	6^3)^	1^3)^	
2000	Bulk carrier US West coast	PH_3_	12	0	
1997	Geared bulker Brazil – Ireland	PH_3_	5	0	

^1)^2 children.

^2)^4 stowaways.

^3)^6 stowaways.

See Table in Additional file 2 for additional cases investigated in detail (Incidents with fumigants/or toxic industrial chemicals. Intoxication cases from 1993–2013).

### Concluding remarks

Both incidents with fumigated containers and bulk cargo ships are scarcely reported in the scientific literature. However, based on the literature survey and the reports referenced above it might seem as if there is a difference with regard to the severity of outcomes in the two cases: So far, there has been only limited reports of fatalities in relation to opening of transport containers (Table 4 and Additional file 2), while fumigation of bulk cargo ships has led to several fatalities, including several stowaways (Table 5). A possible explanation may be that the exposure on a bulk cargo ship may be of considerable longer duration, and that it is more difficult to escape. In addition, the fumigants may be present in the living compartments on the ship without recognised until the exposure has become fatal.

It is important to emphasise that the numbers of incidents reported in Tables 4 and 5 (and the Table in Additional file 2) may be highly underestimated since input from several representatives from both research institutions and national regulatory bodies suggest that a lot of near-accidents and intoxications with serious outcomes are never reported in public. This view was also supported by several presentations and participants at the Berlin workshop in May 22-23^rd^, 2014 (http://www.eomsociety.org/index.php/meetings).

The literature surveys add to this and illustrate one of the major challenges pursuing this field: providing adequate and sufficient documentation of incidents relating to fumigated transport containers including adverse health effects, clinical symptoms, and well documented exposure data, and to make these data public available in well-respected sources.

## Regulations on fumigants

Contemporary trans-continental shipping of consumer goods largely uses tropical softwood to manufacture short-lived or disposable shipping boxes and pallets (wood packaging materials). These items can easily host and transport tropical parasites to other geographical areas, where these foreign organisms often thrive in the absence of their natural predators, and can exert considerable damage to endemic flora, wild animals, cultivated crops and reared animals in temperate areas. This phenomenon is at least as old as the mid-1800 trade of European wine in wooden casks to Northern America. The scarcity of European cask-quality oak forced to manufacture wine casks in the USA, by using local varieties of oak, which carried to Western continental Europe their parasites, mainly *Phyloxera* and *Peronospera*. These foreign parasites quickly expanded in the continent, essentially wiping out most local vine varieties and leading to the treatment of the surviving vineyards with early pesticides, such as the copper-based Bordeaux mixture and later lead and copper arsenates.

The concern that such events may repeat led to discuss at the 6th Conference of the Food and Agriculture Organization of the United Nations (FAO), and to establish in 1952 an international agreement, the International Plant Protection Convention (IPPC), aiming to protect cultivated and wild plants by preventing the introduction and spread of pests. The number of signatory Countries is currently of 181, and the Food and Agriculture Organization of the UN provides the Secretariat of the IPPC. The International Standards for Phytosanitary Measures (ISPMs) are recognized by the World Trade Organization (WTO) for managing pest risks associated with trade, and cover the movement of pests and apply to vehicles, ships, aircraft, containers, storage places, soil, wood packaging and other objects that could harbour plant pests. More than 50 ISPMs, as of 2012, cover issues such as plant quarantine and international trade; pest risk analysis; pest free areas; wood packaging material in international trade; and inspection protocols. Among the technical interventions to counter the spread of pests is the preventive treatment of goods and materials by thermal, irradiation and chemical means. The treatment of materials and goods with volatile chemical agents is referred to as fumigation.

The major fumigant that is subject to regulations at national or to regulated agreements at the international level is methyl bromide. This low-boiling liquid has been used since the late 1930s to protect from spoilage grains shipped in the trans-oceanic routes, as well as in storage silos and in processing facilities, such as in wheat mills, and as a pesticide for greenhouses and for the tilled land. In the late 1980s, it was found that the far-UV absorbing layer of ozone in the upper stratosphere of Antarctica was rapidly vanishing, and therefore the narrowing of the ozone layer in the upper atmosphere above the planetary boundary layer would increase the intensity of carcinogenic far-UV radiation on the Earth and thus have significant deleterious effects on human health. Along with several other synthetic chemicals, such as chloro-fluoro-carbons (CFCs) that were widely used in several fields of technology and of everyday life in consumer products, methyl bromide was identified as an ozone layer-depleting substance. Anticipated by talks and negotiations that started in 1981, a convention was agreed within the UN as early as 1985 to gradually limit, substitute and ban such substances from industrial uses. The Vienna Convention for the Protection of the Ozone Layer is the first treaty ever to achieve universal participation by 1988, and is often called a framework convention, because it served as a framework for efforts to protect the globe’s ozone layer. This Convention paved the way to the later Montreal Protocol on Substances that Deplete the Ozone Layer, which was agreed on September 16, 1987, and entered into force on January 1, 1989. The Montreal Protocol includes a unique mechanism that enables to respond quickly to new scientific information and to accelerate the reductions required on chemicals that are already covered by the Protocol, since these adjustments are then automatically applicable to all countries that ratified the Protocol. Central to the Montreal Protocol is the definition of lists of chemical substances that are recognized to have ozone-depleting characteristics and of programs to phase-out their production and use.

In particular, for methyl bromide there was a need to balance the consequences of two equally binding international treaties, each of which addresses a crucial issue of planet stewardship. Therefore, for methyl bromide a specific exemption was authorized for the amounts used for quarantine and pre-shipment applications (Article 2H: Methyl bromide; para. 6) and a specific identification of the substance in the treaty was provided (Annex E). In addition, the original agreement of the Montreal Protocol allowed a large group of 147 developing countries (Article 5(1) Parties) a slower path to reduction of production, of use and to final ban of several of the controlled substances. The phase-out agreement is articulated into three steps: the first one defining a base level of production, the second a freeze of production at the base level amounts, the third a stepwise gradual reduction in the amounts used, to finally reach 100 per cent reduction of use or its effective cease at a specified date. All uses of methyl bromide by all countries should cease by January 1, 2015. The phase-out schedules of the original agreement and of that following the Adjustments agreed in 1999 in Beijing at the Eleventh Meeting of the Parties are reported in the Tables below (see Tables 6 and 7), as taken from the official UNEP Ozone Secretariat website (http://ozone.unep.org/en/).

**Table 6:** **Summary of control measures under the Montreal Protocol - Annex E - Group I: Methyl bromide**

***Non-Article 5(1) Parties***		***Article 5(1) Parties (developing countries)***	
Base level:	1991	Base level:	Average of 1995-98
Freeze:	January 1, 1995.	Freeze:	January 1, 2002.
25 per cent: reduction	January 1, 1999.	20 per cent: reduction	January 1, 2005.
50 per cent: reduction	January 1, 2001.	100 per cent: reduction	January 1, 2015 (with possible critical use exemptions).
70 per cent: reduction	January 1, 2003.		
100 per cent: reduction	January 1, 2005 (with possible critical use exemptions).		

Applicable to production and consumption, amounts used for quarantine and pre-shipment applications exempted.

**Table 7:** **Allowance for production to meet the basic domestic needs of Article 5(1) Parties following the Beijing Adjustments**

***Non-Article 5(1) Parties***		***Article 5(1) Parties***
Base level:	Production in 1991.	No additional production allowed for basic domestic needs.
January 1, 1995	10 per cent of base level until July 28, 2000.	
New base level for basic domestic needs (effective July 28, 2000)	Annual average production for basic domestic needs of Article 5(1) Parties for the period 1995 to 1998 inclusive.	
July 28, 2000	15 per cent of base level until January 1, 2002.	
January 1, 2002	100 per cent of base level.	
January 1, 2005	80 per cent of new base level.	
January 1, 2015	Zero.	

Annex E - Group I: Methyl bromide.

Other major authorized fumigants are phosphine and phosphine-generating chemicals (metal phosphides) and sulphuryl difluoride. To protect the health of workers at all steps of the maritime logistic chain, a number of bodies at the international and national levels have issued regulations that cover a number of different issues, including the use of hazardous chemicals, such as pesticides and fumigants.

The framework international agreement is the United Nations IMO Safety of Life at Sea (SOLAS) Convention that stems from the first, never enacted agreement of 1914. It was only as late as 1960 that the long sought for UN International Maritime Organization started to develop and maintain a comprehensive regulatory framework for shipping that includes maritime security, safety and environmental concerns, enacting obligation on all Governments to ensure all activities on ships showing their flag are carried out safely. The SOLAS Convention specifically deals with shipping of hazardous items in two of its Chapters, VII and IX. Chapter VII – Carriage of dangerous goods requires that transport of all kinds of dangerous goods comply with the International Maritime Dangerous Goods Code (IMDG Code). Chapter IX - Management for the Safe Operation of Ships, establishes as mandatory the International Safety Management (ISM) Code, which in turn requires the ship owner to establish a safety management system and to empower the ship’s Master to maintain the security of the ship without constraints by the Company, the charterer or any other person.

The IMO further issued several Codes and Recommendations aimed at specific issues. The IMO IMDG Code is the international guideline for the safe transportation or shipment of dangerous goods or hazardous materials by water and is aimed at protecting crewmembers and at preventing marine pollution when hazardous materials are transported on water vessels. The IMO Recommendations on the Safe Use of Pesticides in Ships (revised 2002) is a guide to all those involved in the use of pesticides and fumigants on ships and to governments to comply with their legal obligations under the SOLAS Convention. The IMO last issued a Revised Recommendation On The Safe Use Of Pesticides In Ships Applicable To The Fumigation Of Cargo Transport Units (MSC.1/Circ.1361 27 May 2010) where a number of active substances, still including methyl bromide and its mixture with carbon dioxide, are listed.

The International Maritime Fumigation Organisation (IMFO) is a private non-profit organization to which companies, which provide maritime fumigation services freely adhere. It was constituted with the objective of enabling member companies to provide levels of treatment in accordance to the Code and the Recommendations. IMFO issued a Code of Practice (COP) as a guidance to fumigators and ships' masters in respect of bagged and bulk cargoes, packaged goods (at: http://www.imfo.com*;*www.imfo.com/IMFO_Code_of_Practice.pdf). The COP recognizes in its Introduction that phosphine is the only fumigant that can be used during shipping, and provides fumigators with specific instructions, labelling and checklists.

### Own requirements of individual countries

Most countries directly require all vessels in their territorial waters to comply with the IMO recommendations. The US and the Canadian Coastguards issue their own regulations and specifications (*e.g.*, for Canada: https://www.tc.gc.ca/eng/marinesafety/bulletins-1997-06-eng.htm) and the European Union has issued regulations for the Fumigation of packaged goods only (freight container fumigation) under Regulation CE 1107/2009 and Directives 2008/40/EC, 2006/39/EC e 2003/68/EC.

## Preventive measures

Lessons must be learned from the presented studies, especially since the fumigants are often colourless and odourless, and dangerous even at low concentrations. Since no reliable indication of the absence of toxic gases in closed import containers exist, each container has to be regarded as potentially hazardous. Therefore, such import containers should be entered only if hazardous airborne exposures are excluded, i.e. only after appropriate measurement of air samples or after appropriate aeration, where forced extraction ventilation was shown to be most effective (which necessitates some redesign of containers, e.g. by a hole in the back for applying the extraction pipe). Forced ventilation rapidly reduces toxic concentrations in the container atmosphere; however off gassing within the following hours or days from the goods, which may have absorbed toxic substances, have to be taken into consideration. Thus ventilation should be on-going in initially contaminated containers during stripping due to such off-gassing from goods.

Measuring gases has to be extended from fumigation to include volatile toxic industrial chemicals arising from the production processes. However, it is important to measure not only directly behind the container door (where concentrations may be low due to leakages), but rather to assess the levels deep inside at the bottom, middle, and top of the container. A new flat lancet facilitates this procedure.

It is difficult to measure all possible toxic substances in the container atmosphere; especially formaldehyde and phosphine are not adequately analysed by many devices. So far, no ideal portable non-expensive device is on the market.

More effectively, alternatives for fumigation (such as heat treatment, use of oxygen-depleted air) should be promoted and introduced and toxic industrial chemicals replaced by harmless substances.

The many still existing knowledge gaps, e.g. potentially structural modifications of food by fumigants and health risks for consumers, have to be taken into consideration by future research projects.

The previously mentioned severe and broad health hazards demonstrate an urgent need of common databases, which comprise all health relevant data of each container from the production site, along the transport line, till the end-user. Due to the globalized economy this has to done on national as well as supra-national scales, providing authorities as well as recipients/audience full access to them.

The first comprehensive preventive measures system – which can be considered to be a “State of the art model” – has been implemented in the harbor of Hamburg. The existing risk assessment system is a part of the daily routine to protect the controlling bodies from incidents associated with all kinds of terrorist or criminal threats in the container air and freight. The concept was developed during 2006 till 2010 on the basis of on-site experiences in cooperation with the Institute of Occupational and Maritime Medicine (ZfAM) and the State labor inspection office (Health & Safety Executive Hamburg). It contains a catalog of the minimal requirements for inspections, analyses and an evaluation system. If a potentially toxic gas group (comprising structurally related compounds) is identified by a screening measuring device, additional mandatory analytical toxicological measurements in a specialized analytical laboratory are required [31].

### Applied preventive measures

Appropriate preventive measures for safe handling and stripping of freight containers rely on several factors such as correct labeling, knowledge and awareness among managers and workers, guidelines for safe procedures, measurements, ventilation and personal protective devices.

### Labelling

The first step for taking preventive action is based on the identification of containers with hazardous content. Although labeling is mandatory with warning signs accompanied with transportation documents specifying the fumigation procedures for fumigated containers, several articles and reports between the years 2002 to 2011 have described violations of these regulations. In a study from Rotterdam [46] only three of 303 randomly selected containers had some kind of warning sticker, although methyl bromide or phosphine were detected in 23% of the containers. In Hamburg in 2006 only 3.6% of the 2113 examined containers carried any form of fumigation hazard warning, but none of these corresponded to those required by the IMDG Code and they mostly consisted of fragments of old, presumably outdated, warning [1]. Residual fumigants were detected in a total of 541 (26%) the containers. None of the containers had valid Dangerous Goods Transport Documentation. Safe Work in Australia found that none of 76 surveyed containers displayed any external notice that they had been fumigated, although methyl bromide was identified in 68% of them [25]. In Gothenburg none of the 101 randomly selected containers were labeled [47] http://ki.se/sites/default/files/2011-1_0.pdf They argued that lack in compliance with the labeling regulations raises serious concerns as warning sign is the first, and perhaps only, message the worker receives that suggests the container atmosphere to be hazardous. A Danish study concluded that in practice, the measures applied to prevent harmful chemical exposures released from transport containers are primarily based on the attempt to identify the containers that carry occupational health risks [48]. Discussions with managers and workers in an Australian study suggested that no systematic assessment of containers took place prior to entry by workers [25]).

### Knowledge and awareness

The employers are obliged to inform the workers on all hazards in their work environment and provide adequate training in health and safety at work. The practice of handling containers was recently investigated in a qualitative study in Denmark based on semi-structured interviews with nine key informants, including managers and health and safety representatives of organizations that handle containers [48]; (for details see chapter IV. Practice of handling transport containers in exporting and receiving countries. Pilot studies in China and Denmark). They concluded that there was limited knowledge among managers, workers and even among occupational health professions about the types of chemicals that can be released from containers.

In Australia informal discussions were held with five experienced managers and 15 workers [25]. Also in this study the numbers of workers who completed the surveys was low, and the authors argued that the results should not be used to make generalizations for broader industry or occupational groups. However, about 70% of the workers had completed specific work health and safety training on unpacking shipping containers. None of them knew much about the risks of fumes in containers but 67% knew a little. The most significant reason for not taking safety precautions was lack of training (33%), followed by lack of awareness that the container atmosphere may contain chemical fumes (29%). Thus, although most workers had received work health and safety training there was still a large degree of uncertainty regarding the risks associated with fumigated containers and their ability to identify such containers.

### Guidelines for safe procedures

In addition to the international and national regulations related to container handling there are also local safety instructions from organizations and from employers (e.g. [48-52]).

In the Danish study the interviewed managers were aware of the existence of relevant international and national regulations but were typically not able to specify them [48]. The interviewees were more familiar with the local instructions but these were reported to give insufficient guidance for several practical challenges of preventing chemical exposures during work with containers.

### Measurements

At present there is no single portable instrument available to detect all types of relevant hazardous substances at sufficient sensitivity. However, different approaches, including direct-reading devices and air sampling for later analysis have been used to detect selections of fumigants and toxic gases in various harbors.

The commercial company EWS in Belgium dealing with fumigation suggested that for ad hoc situations handhold technology could be used. Examples of those technologies are [50]:

- Photo ionization detector (PID) for volatile organic compounds (VOC)
- Infrared (spectra) only for sulfuryl difluoride
- Sensors (CO, CO_2_, O_2_, PH_3_)
- Colorimetrical gas detector or indicator tubes, mainly for methyl bromide + 1,2-dichloroethane, benzene, toluene, chloropicrin, styrene, xylene.

Further, measurements for oxygen depletion are recommended.

Onsite measurements/samples from unopened containers should preferably be taken at the middle or top of the container and not only at the bottom of the door where concentrations are lowest [51]. The openings in the top corners of the containers and possible leaking rubber seals around the doors may explain the observation of uneven concentrations in the container. Recently a new flat lancet was developed to facilitate onsite measurements from unopened containers. The lancet was further connected to a direct-reading instrument with detectors for VOCs, formaldehyde, hydrogen cyanide, phosphine and carbon monoxide [51].

In Hamburg, measurements were performed onsite with a gas detector array instrument (GDA, Airsense) by sampling through the online probe inserted into the container. After the measurements with the hand held instrument, special 1 liter air bags were filled with the air of the containers. The air bags were transported to the laboratory for analysis with gas chromatography–mass spectrometry (GC-MS), which requires a well-equipped lab and a well-trained lab technician; in addition, it is time-consuming [5,19,52,53].

Svedberg & Johanson [47] described that on arrival to Sweden, the fumigated and labeled containers are normally handled as all other containers, except when the Swedish Food Agency has border control campaigns during which about 20 containers per year are controlled by measurements. They also reported that as far as they knew most of the central stores and end costumers in Sweden do not have systems to control and handle potentially fumigated/gassed containers, although good examples exist. One large storehouse had a Fourier Transform Infrared--instrument in the central unit. However, they claimed that it is not realistic to require every costumer handling containers, small-sized terminals in particular, to have expensive measurement instruments, but rather rely on sufficient ventilation before entry.

Based on interviews with nine key informants in the Danish study, equipment for monitoring of container air is available in some of the selected organizations only, and even when available, the measuring device can detect only methyl bromide but no other potentially harmful chemicals [48]. In some of these organizations a suspicion is sufficient for applying specific preventive measures while others would require positive results of air measurements to take action.

### Ventilation

The container should be efficiently ventilated before opening when high concentrations of harmful substances have been detected or when measurements have not been done.

Containers normally have small openings in the top corners to provide limited natural ventilation. Svedberg & Johanson [47] evaluated different ventilation methods by tracer gas, and reported that natural ventilation (open doors) and blowing ventilation (open doors, fan blowing air towards goods) had virtually no impact on gas levels in deep container air 12 m from the doors. In contrast, forced extraction ventilation (fan sucking air via a tube inserted all the way into the container and fresh air entering via the doors) resulted in rapid washout of the gas. They concluded that unfortunately the current container design makes safe and speedy sampling and ventilation prior to opening the doors technically difficult. Ventilation must preferably be ongoing during stripping, and a ventilated container that is closed for stripping the following day must be re-ventilated.

In the Australian study [25] the containers were often left to ventilate naturally. The also concluded that for those containers with known high levels of fumigants, natural ventilation may require supplementation with forced ventilation to reduce the concentrations of residual chemicals to acceptable levels for unloading. Industry representatives expressed concern that ventilation systems for extracting fumigants from containers were not effective because the levels of fumigants within containers simply rose again after termination of ventilation and close up of the containers. Thus, it may be useful to set a time limit (e.g. 2 hours) after which unloading should be stopped and the container would have to be ventilated again.

The workers that performed stripping of containers in the Swedish study expressed that the containers frequently carried unpleasant odors that from time-to-time prevented stripping. Such containers were left for natural ventilation before re-entry [47].

The interviewed personnel in the Danish study reported that the main preventive measure to reduce chemical exposure is natural ventilation; active ventilation is not used in practice [48]. The conditions of aeration were not consistently applied; the reported ventilation times varied between 2 and 48 hours without any knowledge of whether this amount of time was sufficient or not.

### Personal protective equipment

In published articles and reports the use of PPE during container stripping has only been briefly mentioned, and it has not been described which type of protective mask or other PPE that have been used. The types of required PPE differ from one toxicant to another and it is emphasized that the correct PPE should be used. In addition, rescue strategies should be available to individuals who need to enter unventilated containers with unknown hazards [47,31].

In the Australian study one-third reported use of PPE, but they did not specify which kind of PPE they used [54]. Knol-de Vos [46] noted that staff wore personal PPE when opening containers in Rotterdam. Pedersen et al. [48] reported that although masks are usually provided to the workers in Denmark, they are mainly dust masks that do not prevent exposure to gaseous chemicals. The also described that the decision and choice of preventive measures to be used in practice are often left to the directly involved employees and the actions taken show considerable variation. When the workers in Sweden experienced unpleasant odors, the containers were left for natural ventilation, or, alternatively, the workers were instructed to wear respiratory equipment [47].

WorkSafe [54] summarizes that consistent with the risk assessment for containers with methyl bromide mixtures, PPE considerations could include:

- Elbow length chemical gloves;
- Full coveralls;
- Full face mask equipped with:
  - ○ multi-gas filters; or
  - ○ an organic vapor cartridge filter, that is designed to cover methyl bromide and chloropicrin
  - ○ mixed gas (filter type will depend upon the brand & type of full face mask that you purchase); or
- Self-Contained Breathing Apparatus (SCBA).

It is important to ensure regular training and instruction about the procedures and the maintenance and use of PPE to ensure that staffs are competent in its use. Records of this training should be maintained.

## Additional files

Additional file 1:
**Questionnaire FumEx2.**
Additional file 2:
**Table “Incidents with fumigants/or toxic industrial chemicals.** Intoxication cases, as reported (1993–2013)”.
