# Correction to: Health risks in international container and bulk cargo transport due to volatile toxic compounds

**DOI:** 10.1186/s12995-018-0207-8

**Published:** 2018-08-13

**Authors:** Xaver Baur, Lygia Therese Budnik, Zhiwei Zhao, Louis Verschoor, Federico Maria Rubino, Claudio Colosio, Jorgen R. Jepsen

**Affiliations:** 1 European Society for Environmental and Occupational Medicine (EOM), Berlin, Germany; 2 Institute for Occupational Medicine, Charité University Medicine, Charité-Campus Benjamin Franklin, Berlin, Germany; 3 Division Occupational Toxicology and Immunology, Institute for Occupational and Maritime (ZfAM), University Medical Center Hamburg-Eppendorf, University of Hamburg, Hamburg, Germany; 4 Dalian Maritime University (DMU), Dalian, Ganjingzi China; 5 Expertise Centre Environmental Medicine (ECEMed), Rijnstate Teaching Hospital, Velp, The Netherlands; 6 Department of Health Sciences of the University of Milano and International Centre of Rural Health, San Paolo University Hospital Milano, Milan, Italy; 7 Centre of Maritime Health and Society, Institute of Public Health, University of Southern Denmark, Odense, Denmark

## Correction

Magne Bråtveit and Rune Djurhuus have removed themselves as authors from this article [1] as they did not approve the final version and they do not agree with the revisions made to the section “Occurrences of intoxications with chemicals used for pest control in transport containers and on bulk cargo ships” and Table 2, including relationships between claimed exposure and health effects and the statement on fatalities related to container opening.

The authorship list for this article is now: Xaver Baur, Lygia Therese Budnik, Zhiwei Zhao, Louis Verschoor, Federico Maria Rubino, Claudio Colosio and Jorgen R Jepsen.

Loius Verschoor, Frederico Maria Rubino and Claudio Colosio did not respond to correspondence about this erratum.
