# Supplementary material for: Health risks in international container and bulk cargo transport due to volatile toxic compounds
Source: J Occup Med Toxicol. 2015 May 20;10:19. doi: 10.1186/s12995-015-0059-4 (PMC4465480; doi:10.1186/s12995-015-0059-4)
Supplement: Additional file 1: — Questionnaire FumEx2. [file 12995_2015_59_MOESM1_ESM.pdf]

Fum-Ex 2

FUMIGANT EXPOSURE QUESTIONNAIRE

STUDY SITE (INSTITUTION) ____

CO-INVESTIGATOR ____

DATE OF EVALUATION ___/___/______

NAME-ACRONYM/or Nr. ____

DATE OF BIRTH ___/___/______ (MONTH/DAY/YEAR) AGE ___ YEARS

SEX F___ M ___

HEIGHT (cm) _______

WEIGHT (kg) _______

CURRENT SMOKER: YES ___ NO ___ NEVER SMOKED ___

EX-SMOKER: YES ___ NO ___

DATE QUIT: ___/___/______ (MONTH/DAY/YEAR)

SMOKING HISTORY IN # OF PACK YEARS: ___ PACK-YEARS

OCCUPATIONAL HISTORY

1. JOB DESCRIPTION ____

   ____

2. SINCE WHEN ARE YOU WORKING IN YOUR CURRENT JOB?
   ___ (M)/ _______ (Y)

3. DO YOU CURRENTLY WORK WITH FUMIGANTS, PESTICIDES OR OTHER CHEMICALS?

   YES ___ NO ___

4. IF YES TO QUESTION # 3, SPECIFY: ___ Methyl bromide

   ___ Ethylene dichloride

   ___ Methylene chloride

   ___ Phosphine

   ___ ______________________________ (OTHER)

   I. DURATION OF EXPOSURE IN TOTAL ______ (MONTHS)
   II. HOW MANY HOURS DO YOU WORK WITH THE AGENTS MENTIONED ABOVE PER WEEK?

___ HOURS

III. WHEN WAS THE LAST EXPOSURE? ___/___/______ (MONTH/DAY/YEAR)

IV. DURATION OF LAST EXPOSURE ____ (DAYS) ____ (HOURS) ____ (MINUTES)

5. IF NO (QUESTION # 3): DID YOU WORK WITH THESE AGENTS IN THE PAST?

YES ___ NO ___

WHICH AGENT? ____

WHAT WAS YOUR JOB DESCRIPTION AT THAT TIME? ___________________________

EXPOSURE STARTED (DATE) ___/______ (MONTH/YEAR)

EXPOSURE ENDED (DATE) ___/______ (MONTH/YEAR)

6. WHILE WORKING DID YOU USE ANY PROTECTION EQUIPMENT? YES ___ NO ___

IF YES: WHICH? ____

7. Did YOU had contact to genotoxic agents?

YES ___ NO ___

WHICH AGENT? ____

8. Have YOU been exposed to ionizing radiation for dignostic purposes?

YES ___ NO ___

How long?____

9. SYMPTOMS

HOW MANY TIMES DID THE FOLLOWING SYMPTOMS OCCUR DURING THE LAST 12 MONTHS?

SYMPTOMS/INCIDENCE	ALMOST ALWAYS	OFTEN	SPORADIC	ALMOST NEVER	NEVER	WHEN DID IT OCCURE FOR THE FIRST TIME? (M/D/Y)	WAS IT WORK-RELATED? YES/NO
HEADACHE	I_I	I_I	I_I	I_I	I_I	___/___/______	
CHEST TIGHTNESS, DYSPNEA	I_I	I_I	I_I	I_I	I_I	___/___/______	
AIRWAYS IRRITATION, COUGH	I_I	I_I	I_I	I_I	I_I	___/___/______	
MUCOSA IRRITATIONS (RHINITIS, STOMATITIS)	I_I	I_I	I_I	I_I	I_I	___/___/______	
EYE IRRITATION, CONJUNCTIVITIS	I_I	I_I	I_I	I_I	I_I	___/___/______	
NAUSEA	I_I	I_I	I_I	I_I	I_I	___/___/______	
DIZZINESS	I_I	I_I	I_I	I_I	I_I	___/___/______	
MUSCLE CRAMPS	I_I	I_I	I_I	I_I	I_I	___/___/______	
CONCENTRATION DISORDERS	I_I	I_I	I_I	I_I	I_I	___/___/______	
DYSGEUSIA	I_I	I_I	I_I	I_I	I_I	___/___/______	
NUMBNESS	I_I	I_I	I_I	I_I	I_I	___/___/______	
DIARRHEA, ABDOMINAL CRAMPS	I_I	I_I	I_I	I_I	I_I	___/___/______	
WEAKNESS, FATIGUE	I_I	I_I	I_I	I_I	I_I	___/___/______	
SEIZURES	I_I	I_I	I_I	I_I	I_I	___/___/______	
DISTURBANCE OF						___/___/______	

DISTURBANCE OF MEMORY	I_I	I_I	I_I	I_I	I_I	___/___/______	
SYMPTOMS/INCIDENCE	ALMOST ALWAYS	OFTEN	SPORADIC	ALMOST NEVER	NEVER	WHEN DID IT OCCURE FOR THE FIRST TIME? (M/D/Y)	WAS IT WORK-RELATED? YES/NO
EMOTIONAL INSTABILITY	I_I	I_I	I_I	I_I	I_I	___/___/______	
SADNESS, DEPRESSION	I_I	I_I	I_I	I_I	I_I	___/___/______	
SLEEP DISORDER	I_I	I_I	I_I	I_I	I_I	___/___/______	
IMPAIRED BALANCE, DISTURBED GAIT	I_I	I_I	I_I	I_I	I_I	___/___/______	
TREMOR	I_I	I_I	I_I	I_I	I_I	___/___/______	
OTHER, PLEASE SPECIFY ____________________	I_I	I_I	I_I	I_I	I_I	___/___/______	

10. HAVE YOU EVER BEEN UNCONSCIOUS IN THE LAST YEARS? YES ___ NO ___
11. IF <u>YES</u> (QUESTION #10): DID IT HAPPEN AT YOUR WORKPLACE? YES ___ NO ___
12. PLEASE INDICATE BELOW WHICH CHRONIC OR ACUTE CONDITION(S) YOU HAVE:
    - I_I ARTHRITIS, SPECIFY ____
    - I_I RHEUMATIC DISEASE, SPECIFY ____
    - I_I ASTHMA, SPECIFY ____
    - I_I CHRONIC BRONCHITIS ____
    - I_I CANCER, SPECIFY ____
    - I_I DIABETES, SPECIFY ____
    - I_I KIDNEY DISEASE, SPECIFY ____
    - I_I LIVER DISEASE, SPECIFY ____
    - I_I CENTRAL/PERIPHERAL NERVOUS SYSTEM DISEASE, SPECIFY _________________________________
    - I_I OTHER CHRONIC CONDITION, SPECIFY ____

      ____
13. ARE YOU CURRENTLY TAKING ANY MEDICATION? YES ___ NO ___

    IF YES, SPECIFY: ____

14. DID YOU HAD CONTACT TO GENOTOXIC AGENTS?

    YES ___ NO ___

    WHICH AGENT? ____

    WHEN? _________
15. HAVE YOU BEEN EXPOSED TO IONIZING RADIATION FOR DIGNOSTICOR THERAPEUTIC PURPOSES?

    YES ___ NO ___

    WHEN?____

    HOW LONG?____

16. DIET

MEAT

HOW MANY TIMES PER WEEK?____________

VEGETABLES

HOW MANY TIMES PER WEEK?____________

FRUITS

HOW MANY TIMES PER WEEK?____________

ALCOHOL CONSUMTION

WINE_________________ BEER________________ HIGH% SPIRITS ________________

HOW MANY TIMES PER WEEK?____________

17. ADDITIONAL INFORMATION IF NEEDED: ____

____

____

THANK YOU VERY MUCH FOR YOUR COOPERATION!

see also :

http://www.eomsociety.org/attachments/FUM-EX%202_%20QUESTIONNAIRE_EOM%20FRENCH%20VERSION.pdf

for the French Version of the Questionnair
