# Supplementary material for: Health risks in international container and bulk cargo transport due to volatile toxic compounds
Source: J Occup Med Toxicol. 2015 May 20;10:19. doi: 10.1186/s12995-015-0059-4 (PMC4465480; doi:10.1186/s12995-015-0059-4)
Supplement: Additional file 2: — Table “Incidents with fumigants/or toxic industrial chemicals. Intoxication cases, as reported (1993–2013)”. [file 12995_2015_59_MOESM2_ESM.pdf]

Health risks in international container and bulk cargo transport due to volatile toxic compounds
Xaver Baur, Lygia Therese Budnik, Zhiwei Zhao, Magne Bratveit, Rune Djurhuus, Louis Verschoor, Claudio Colosio, Jorgen Jepsen,

Incidents with fumigants/ or toxic industrial chemicals. Intoxication cases (1993-2013)

R	Exposure			Main Complaints	Diagnosis
	Substance	Activity	Period		
1	1,2-Dichloroethane	Unloading a Container	4 - 5 hours	Headache, vertigo, nausea, dyspnoea, pruritus, loss of strength in left hand	• Intoxication with fumigants<br>• Pulmonary obstruction and bronchial hyperresponsivness (RADS)<br>• Persistent neurological deficiencies
2	1,2-Dichloroethane	Unloading a Container	Ca. 6 hours	Headache, vertigo, nausea, dyspnoea, visual disturbances, metallic taste, chest pain	• Intoxication with fumigants<br>• Pulmonary obstruction and bronchial hyperresponsivness (RADS)
3	1,2-Dichloroethane	Unloading a Container	Ca. 4 + 2 hours	Headache, vertigo, nausea, dyspnoea, burning skin sensation, metallic taste, furred tongue, attacks of sweating	Intoxication with fumigants
4	1,2-Dichloroethane	Unloading a Container	Approx. 4 hours	Headache, nausea, furred tongue, dry mouth, sleep disorder, bloated feeling, flatulence	Intoxication with fumigants
5	1,2-Dichloroethane	Unloading a Container	Approx. 10 minutes	Headache, dizziness, nausea, burning skin sensation, furred tongue, burning sensation and numbness of the lips	• Intoxication with fumigants<br>• Bronchial hyperresponsivness<br>• Intermittent atrial fibrillation and absolute arrhythmia<br>• Hyperlipidemia
6	Phosphine	Storage room	Ca. 30 minutes	Headache, lassitude, nervousness, airway irritation, cough, burning of facial skin, itching conjunctivitis , taste disorder, anaesthesia of left hand	• Phosphine intoxication<br>• Persistent polyneuropathy<br>• Persistent cerebral dysfunction<br>• Bronchial hyperresponsivness

Note : <u>the table is not complete</u>, since many cases are not published, the dark numbers reported to the insurances cannot be estimated (data is not available). In each case mostly several persons were intoxicated, the exact number of exposed persons were not always provided, however.

Incidents with fumigants/ or toxic industrial chemicals. Intoxication cases (1993-2013)

7	1,2-Dichloroethane	Warehouse (Storing textiles)	2 years	Headache, vertigo, nausea, dyspnoea, impaired fitness, disturbance of concentration and memory, nasal and oral complaints	• Intoxication with fumigants<br>• Persistent cerebral dysfunction<br>• Bronchial hyperresponsivness
8	1,2-Dichloroethane	Storing textiles in a warehouse	10 years	Headache, vertigo, nausea, dyspnoea, motoric disorder, rhinorrhoea, tightness in the chest	• Intoxication with fumigants<br>• Persistent disturbance of memory and concentration disorders<br>• Bronchial hyperresposivness
9	1,2-Dichloroethane	Cleaning warehouse	4 years	Headache, cognitive disorder, disturbance of concentration, emotional instability, airways irritation and cough	• Intoxication with fumigants<br>• Type-I-sensitisation versus tree and weed
10	unknown	Container inspection	some minutes	Headache, vertigo, nausea, dyspnoea, visual disturbances, disturbance of memory and concentration disorders	• Intoxication with fumigants
11	Methyl bromide	Unloading Container	5-6 minutes	Headache, dyspnoea, cough, pruritus, repetitive epistaxis, reduced fitness, fatigue, muscle cramps, concentration disorders, nervousness	• Intoxication with fumigants<br>• Bronchial hyperresponsivness
12	unknown	Storing textiles in a warehouse	4 years	Dyspnoea, cough, red and watering eyes, runny nose, sneezing, temperature, exhaustion	• Intoxication with fumigants<br>• Type-I-sensitisation versus cats, dogs, weeds, goats and guinea-pigs
13	Methyl bromide	Loading and Unloading of Containers	8 years	Dyspnoea, vertigo, metal taste, red and itching eyes, abdominal pain	• Intoxication with fumigants

Note : <u>the table is not complete</u>, since many cases are not published, the dark numbers reported to the insurances cannot be estimated (data is not available). In each case mostly several persons were intoxicated, the exact number of exposed persons were not always provided, however.

Incidents with fumigants/ or toxic industrial chemicals. Intoxication cases (1993-2013)

14	Methyl bromide, Phosphine	Unloading Containers, Storing goods (warehouse)	9 years	Headache, retinal bleeding, impaired vision	• Methyl bromide intoxication<br>• Phosphine intoxication
15	Dichloromethane	Moving own furniture via Container from South America to Europe	weeks	Headache, dizziness, irascibility, bitter taste, burning nose, tongue and airways, cough, cognitive disorder, disturbance of concentration, anomic aphasia,, fatigue , muscle cramps and aching muscles	• Dichloromethane intoxication
16	Methyl bromide	Unloading of containers	1 year	Headache, mucosa and skin irritations, nausea, dizziness, concentration disorders, fatigue, slurred speech, muscle cramps	• Intoxication with methyl bromide<br>• Prostate carcinoma
17	Methylene chloride Dichloromethane	Ironing imported textiles	2 years	Airways irritation, cough, chest tightness, dyspnea	• Methylene chloride intoxication<br>• Bronchial hyperresponsivness
18	unknown	Container inspection	1 hour	Headache, nausea, dizziness, irritation of hand and facial skin, airways irritation	• Intoxication with fumigants<br>• Headache, Nausea, Dizziness
19	Methylene chloride	Ironing imported textiles, Storing textiles	2 years	Dizziness, weakness, fatigue, chronic fatigue and reduced physical capacity	• Methylene chloride intoxication<br>• Bronchial hyperresponsivness

Note : the table is not complete, since many cases are not published, the dark numbers reported to the insurances cannot be estimated (data is not available). In each case mostly several persons were intoxicated, the exact number of exposed persons were not always provided, however.

Incidents with fumigants/ or toxic industrial chemicals. Intoxication cases (1993-2013)

20	Phosphine	Container inspection	1 day	Headache, airways irritation and dyspnea, cough, nausea, dizziness, concentration disorders and disturbance of memory, chronic fatigue and/or reduced physical capacity, emotional instability, muscle cramps, slurred speech, parasthesia, sleep disorder	• Phosphine intoxication<br>• Bronchial hyperresponsivness (RADS)
21	Methyl bromide	Unloading of containers	2 years	Headache, airways irritation, mucosa irritations, dizziness, emotional instability, muscle cramps	• Methyl bromide intoxication<br>• Bronchial hyperresponsivness (RADS)
22	Methyl bromide	Unloading of containers	3 years	Headache, nausea, dizziness, dysgeusia	• Methyl bromide intoxication
23	Methyl bromide	Unloading of containers	2 years	Headache, airways irritation and cough, chest tightness, dyspnea, skin irritation, muscle cramps, chronic fatigue and/or reduced physical capacity, emotional instability, dysgeusia	• Methyl bromide intoxication<br>• Bronchial hyperresponsivness
24	Methyl bromide	Unloading of containers	6 months	Headache, airways irritation, mucosa irritation, disturbance of memory, concentration disorders, chronic fatigue and/or reduced physical capacity, emotional instability	• Methyl bromide intoxication

Note : <u>the table is not complete</u>, since many cases are not published, the dark numbers reported to the insurances cannot be estimated (data is not available). In each case mostly several persons were intoxicated, the exact number of exposed persons were not always provided, however.

Incidents with fumigants/ or toxic industrial chemicals. Intoxication cases (1993-2013)

25	Methyl bromide	Unloading of containers	1 year	Headache, mucosa irritation, concentration disorders, disturbance of memory, chronic fatigue and/or reduced physical capacity	• Methyl bromide intoxication
26	Phosphine	Unloading of containers	2 months	Headache, airways irritation, cough, skin irritations, nausea, dizziness, muscle cramps, diarrhea, abd. cramps	• Phosphine intoxication
27	Phosphine	Unloading of containers	4 years	Headache, airways irritations, cough, mucosa irritations, nausea, muscle cramps, diarrhea, abd. cramps, chronic fatigue and/or reduced physical capacity, dizziness	• Phosphine intoxication
28	Methyl bromide	Unloading of containers	1 day	Headache, airways irritations, cough, skin and mucosa irritations, nausea, dizziness	• Methyl bromide intoxication
29	Methyl bromide	Unloading of containers	1 day	Skin and mucosa irritations, dizziness, muscle cramps, concentration disorders, dysgeusia, numbness, seizures, disturbance of memory, chronic fatigue and/or reduced physical capacity, chest tightness, dyspnea, emotional instability, slurred speech, paraesthesia, sleep disorder	• Methyl bromide intoxication
30	1,2-Dichloroethane	Logistic company		Disturbance of consciousness, rapid breathing, convulsions	• 1,2-Dichloroethane intoxication

Note : the table is not complete, since many cases are not published, the dark numbers reported to the insurances cannot be estimated (data is not available). In each case mostly several persons were intoxicated, the exact number of exposed persons were not always provided, however.

Incidents with fumigants/ or toxic industrial chemicals. Intoxication cases (1993-2013)

31	Dichloromethane, 1,1,1-trichloroethane, toluene, ethyl acetate, MEK	Adhesive work		Decrease of muscle strength, muscle atrophy in the hands	• Chronic polyneuropathy due to dichloromethane
32	Methyl bromide	Fumigation	1 hour	Dizziness, fatigue, nausea and vomiting, chest pain, dyspnoea, ataxia, general convulsions, anuria	• Methyl bromide intoxication
33	Methyl bromide	Fumigation of grain store	1 hour	Dizziness, fatigue, nausea and vomiting, chest pain, dyspnoea	• Methyl bromide intoxication
34	Methyl bromide	fumigated house	1 day	Headache, status epilepticus, hypoglycaemia, rhabdomyolysis, heart with areas of oedema and focal haemorrhage, severe lung oedema, bilateral hippocampal necrosis, death	• Methyl bromide intoxication
35	Methyl bromide	Working in warehouse of imported and fumigated fruit and vegetable	Several hours	Paresthesia in the feet, unstable gait, blepharoptosis, pharyngeal pain,	• Methyl bromide intoxication
36	Methyl bromide	Fumigation	9 hours	Feeling sick	• Methyl bromide intoxication
37	Methyl bromide	Fumigation	30 hours	Impaired consciousness, convulsions	• Methyl bromide intoxication
38	Methyl bromide	Fumigation	30 Hours	Impaired consciousness, convulsions	• Methyl bromide intoxication

Note : <u>the table is not complete</u>, since many cases are not published, the dark numbers reported to the insurances cannot be estimated (data is not available). In each case mostly several persons were intoxicated, the exact number of exposed persons were not always provided, however.

Incidents with fumigants/ or toxic industrial chemicals. Intoxication cases (1993-2013)

39	Phosphine	Fumigation at sea	Hours - Days?	Death	• Phosphine intoxication
40	Phosphine	Aluminium phosphide manufacturing	Hours	Chest pain, dyspnoea, dizziness, nausea and vomiting	• Phosphine intoxication
41	Methyl Bromide	Cleaning a grain silo	Minutes	Impaired consciousness, death the same night	• Methyl bromide intoxication
42	unknown	Opening ship´s hold after fumigation of lumber	Minutes	Death	• Fumigant intoxication<br>• Anoxia
43	unknown	Ship´s inspection	Minutes	Death	• Fumigant intoxication
44	Phosphine	Transportation of fumigated cargo on ship	Several Days	Death (2 cases)	• Death by Phosphine intoxication (2 cases)
45	Phosphine	Transportation of fumigated cargo on ship	Several Days	6 „poisoned" seafarers	• 6 cases of Phosphine intoxication
46	Phosphine	Grain fumigation on board	Several Days	Group of ship´s crew „poisoned"	• Group Phosphine intoxication
47	Phosphine	Stowaway in Ship´s hold with fumigated cargo	49 Hours	When found: unconscious, weak pulse, CPR necessary. 45 minutes later: dead	• Death due to Phosphine Intoxication
48	Phosphine	fumigated cargo	2 days	„poisined"	• Phosphine Intoxication
49	Phosphine	Investigation of clandestine meth-amphetamine laboratories	20-30 Minutes	Initially: Dizziness, dry cough, headache, diarrhea Persistent: dry cough	• Phosphine Intoxication

Note : <u>the table is not complete</u>, since many cases are not published, the dark numbers reported to the insurances cannot be estimated (data is not available). In each case mostly several persons were intoxicated, the exact number of exposed persons were not always provided, however.

Incidents with fumigants/ or toxic industrial chemicals. Intoxication cases (1993-2013)

50	Methyl bromide,	Fumigation of a museum	28-30 Hours	Feeling sick after about 10-12 hours of exposure, dead after 28-30 hours of exposure	• Intoxication with methyl bromide
51	Ethylene oxide	Fumigation of a museum	several hours	Tremors of the hand, later myoclonus, coma, generalized myoclonic convulsions Persistent: mild tremor and intention tremor of right hand	• Intoxication with ethylene oxide
52	Methyl bromide, ethylene oxide	Fumigation of products (inhala-tion of gas when removing seal of cylinder without wearing gas mask)	Seconds	Initially: mild nausea and vomiting. After 3 days dizziness, light-headedness, allophasis, abasia, decreased muscle strength in upper and lower extremities, glove and stocking anaesthesia	• Intoxication with methyl bromide and ethylene oxide
53	Sulfuryl Fluoride	Fumigation of condominium	Several Hours	Death	• Death due to sulfuryl fluoride intoxication
54	Methyl bromide	Unpacking of import goods in a storage room	several hrs	mucosal, skin, and eye irritation, once bleeding nose , neurological symptoms (nausea, headache)	• Intoxication with methyl bromide
55	Ethylene oxide	Unpacking of import goods in a storage room	several hrs	mucosal, skin, and eye irritation, once bleeding nose , neurological symptoms (nausea, headache)	• Intoxication with ethylene oxide

Note : <u>the table is not complete</u>, since many cases are not published, the dark numbers reported to the insurances cannot be estimated (data is not available). In each case mostly several persons were intoxicated, the exact number of exposed persons were not always provided, however.
